# Supplementary material for: Indirect Treatment Comparison of First-Line CDK4/6-Inhibitors in Post-Menopausal Patients with HR+/HER2− Metastatic Breast Cancer
Source: Cancers (Basel). 2023 Sep 14;15(18):4558. doi: 10.3390/cancers15184558 (PMC10527344; doi:10.3390/cancers15184558)
Supplement: Supplementary file 1 [file cancers-15-04558-s001.zip › cancers-2539033-supplementary.pdf]

Supplementary Materials

Indirect Treatment Comparison of First-Line CDK4/6-Inhibitors in Post-Menopausal Patients with HR+/HER2– Metastatic Breast Cancer

Joseph J. Zhao, Khi Yung Fong, Yiong Huak Chan, Jeremy Tey, Shaheenah Dawood, Soo Chin Lee, Rich-ard S. Finn, Raghav Sundar and Joline S. J. Lim

Table S1. Full search phrases for respective databases.

**PubMed** 821 articles
(breast OR ductal) AND (cancer OR carcinoma) AND (metasta* OR disseminat* OR unresectable OR widespread OR advanced) AND (CDK OR 'cyclin-dependent kinase' OR aromatase OR ribociclib OR abemaciclib OR palbociclib) AND random*
**Embase** 153 articles
('breast'/exp OR breast OR ductal) AND ('cancer'/exp OR cancer OR 'carcinoma'/exp OR carcinoma) AND (metastatic OR 'metastases'/exp OR 'metastasis'/exp OR disseminated OR unresectable OR widespread OR advanced) AND (cdk OR 'cyclin-dependent kinase'/exp) AND (random OR randomized OR randomised) NOT ('animal'/exp NOT 'hu-mans'/exp) NOT ('systematic review'/de OR 'meta analysis'/de OR [editorial]/lim OR [erratum]/lim OR [note]/lim OR [review]/lim OR [short survey]/lim)

Date searched: 15th September 2022.

Table S2. Comparisons of reconstructed curves and original curves.

**Trial, out-come**	**Original**	**Reconstructed**
**MON-ALEESA2 Overall Sur-vival**	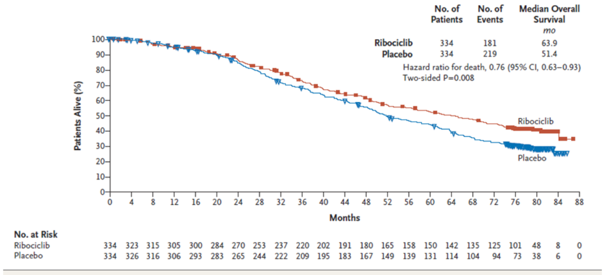Figure 1. Overall Survival. Patients in both groups also received letrozole. Squares (ribociclib group) and triangles (placebo group) indicate censored data.	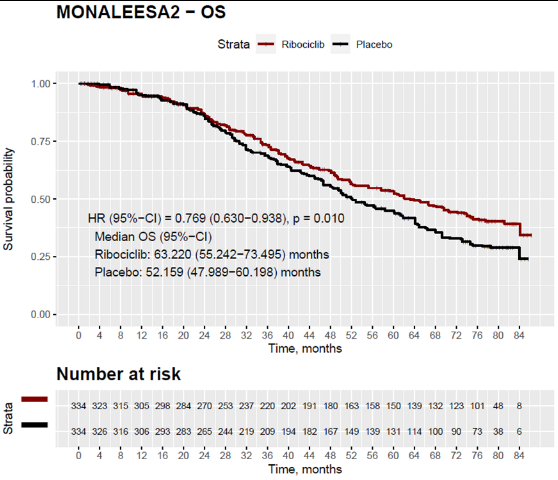

MONALEESA2 Progression-Free Survival

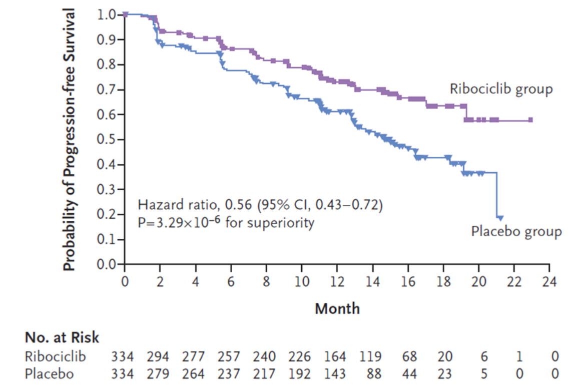

Figure 1. Kaplan–Meier Analysis of Progression-free Survival. After 18 months, the progression-free survival rate was 63.0% (95% CI, 54.6 to 70.3) in the ribociclib group and 42.2% (95% CI, 34.8 to 49.5) in the placebo group. The median duration of progression-free survival was not reached in the ribociclib group and was 14.7 months in the placebo group.

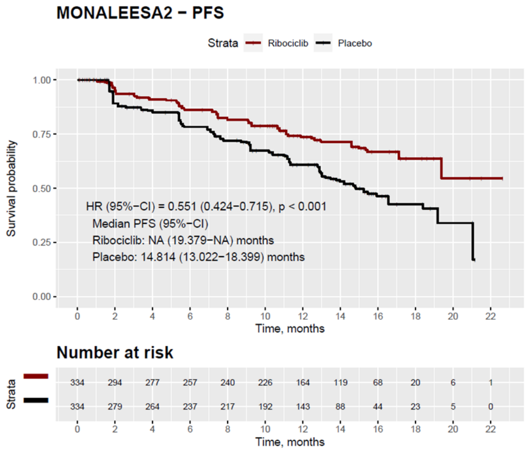

MONARCH 3 Overall Survival

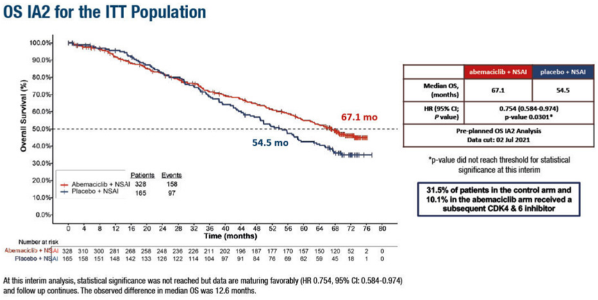

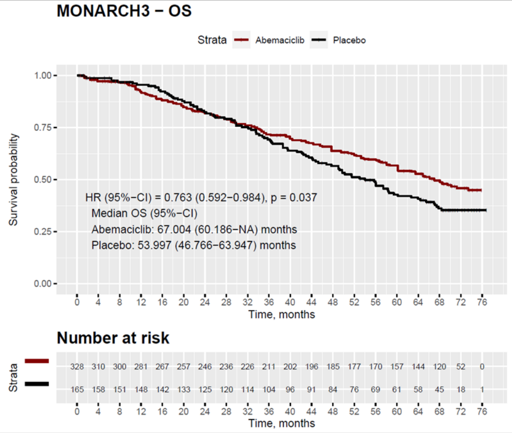

MONARCH 3 Progression Free Survival

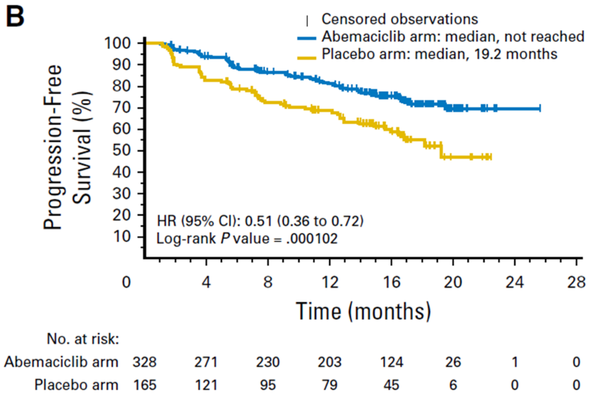

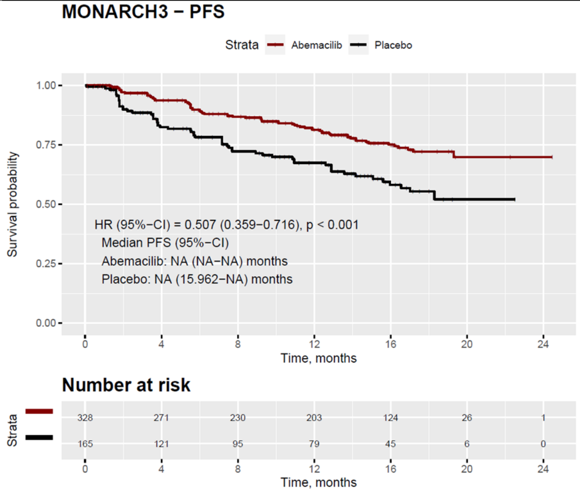

PALOMA 2 Overall Survival

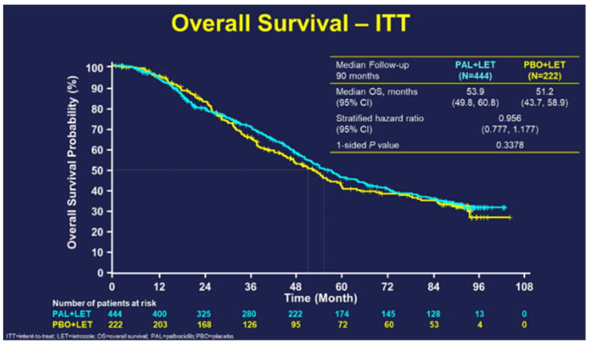

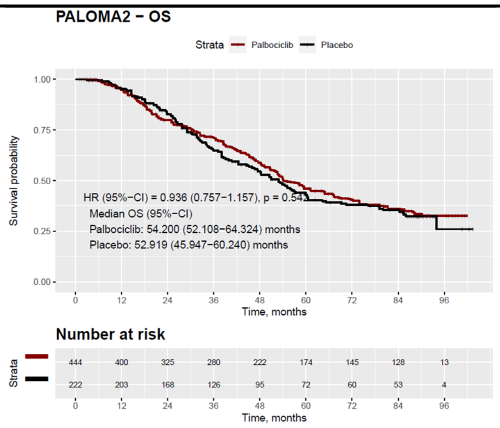

PALOMA 2 Progression Free survival

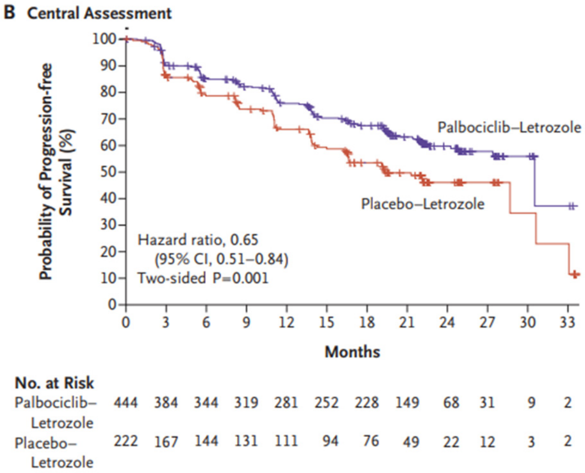

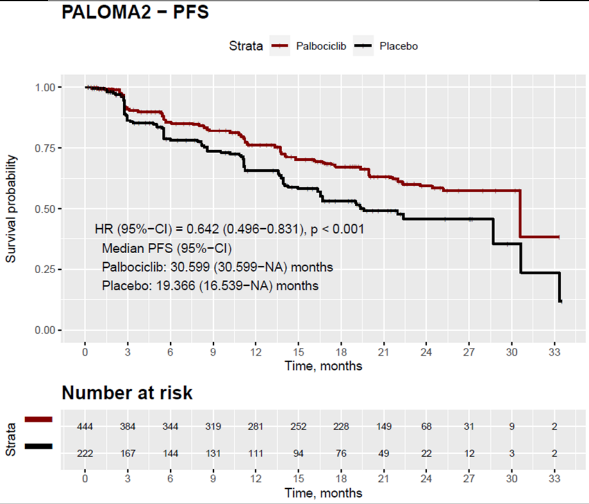

Table S3 Comparisons of reconstructed curves and original curves.

Comparison	Study design	Risk of bias	Inconsistency	Indirectness	Imprecision	Effect (95%CI)	Certainty
Palbociclib vs ribociclib (OS)	RCT	Not serious	Not serious	Not serious	Not serious	0.914 (0.689-1.212)	⨁⨁⨁⨁ High
Palbociclib vs abemaciclib (OS)	RCT	Not serious	Not serious	Not serious	Not serious	1.265 (0.934-1.714)	⨁⨁⨁⨁ High
Abemaciclib vs ribociclib (OS)	RCT	Not serious	Not serious	Not serious	Not serious	0.722 (0.520-1.002)	⨁⨁⨁⨁ High
Palbociclib vs ribociclib (OS)	RCT	Not serious	Not serious	Not serious	Not serious	1.107 (0.914-1.341)	⨁⨁⨁⨁ High
Palbociclib vs abemaciclib (OS)	RCT	Not serious	Not serious	Not serious	Not serious	1.187 (0.971-1.450)	⨁⨁⨁⨁ High
Abemaciclib vs ribociclib (OS)	RCT	Not serious	Not serious	Not serious	Not serious	0.933 (0.753-1.157)	⨁⨁⨁⨁ High

OS, overall survival; RCT, randomized controlled trial; CI, confidence interval.

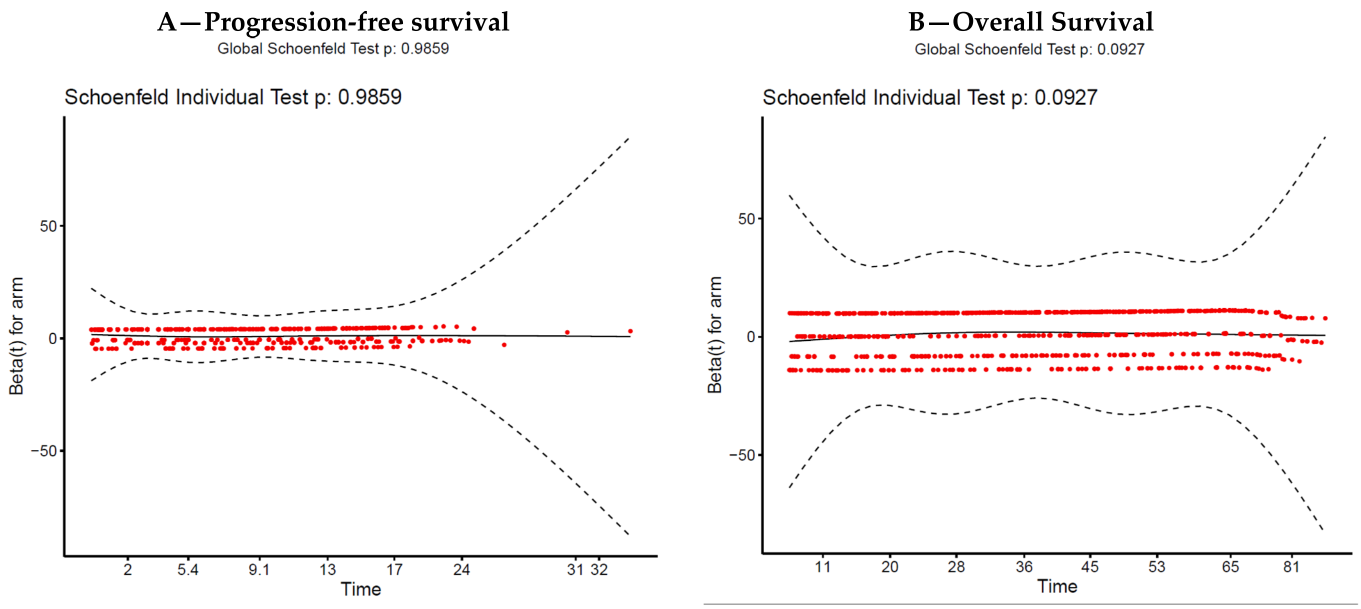

Figure S1. Assessment of proportional hazards assumption.

	Comparison	One-Stage HR (95%-CI)	One-Stage p-value	Two-Stage HR (95%-CI)	Two-Stage p-value
A Progression-free survival	ribociclib vs palbociclib	1.094 (0.825 – 1.451)	0.531	0.858 (0.594 – 1.239)	0.415
	abemaciclib vs palbociclib	0.790 (0.583 – 1.071)	0.129	0.790 (0.514 – 1.216)	0.285
	placebo vs palbociclib	1.745 (1.419 – 2.146)	<0.001	1.559 (1.204 – 2.018)	<0.001
	abemaciclib vs ribociclib	0.722 (0.520 – 1.002)	0.051	0.921 (0.597 – 1.420)	0.710
	placebo vs ribociclib	1.595 (1.255 – 2.027)	<0.001	1.816 (1.398 – 2.360)	<0.001
	placebo vs abemaciclib	2.209 (1.688 – 2.891)	<0.001	1.972 (1.396 – 2.785)	<0.001
B Overall survival	ribociclib vs palbociclib	0.903 (0.746 – 1.094)	0.297	0.821 (0.614 – 1.098)	0.183
	abemaciclib vs palbociclib	0.843 (0.690 – 1.030)	0.094	0.815 (0.585 – 1.135)	0.227
	placebo vs palbociclib	1.124 (0.963 – 1.312)	0.137	1.068 (0.864 – 1.320)	0.542
	abemaciclib vs ribociclib	0.933 (0.753 – 1.157)	0.528	0.993 (0.719 – 1.372)	0.966
	placebo vs ribociclib	1.245 (1.046 – 1.482)	0.014	1.301 (1.066 – 1.588)	0.010
	placebo vs abemaciclib	1.334 (1.111 – 1.603)	0.002	1.310 (1.016 – 1.690)	0.037

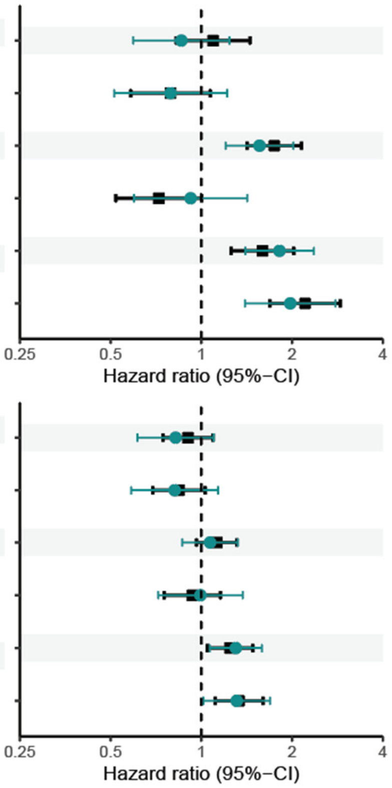

Figure S2. Interval plots – with comparisons against placebo; R, hazard ratio; CI, confidence interval.
